# The HIV/AIDS epidemic in sub-Saharan Africa: thinking ahead on programmatic tasks and related operational research

**DOI:** 10.1186/1758-2652-14-S1-S7

**Published:** 2011-07-06

**Authors:** Rony Zachariah, Wim Van Damme, Vic Arendt, Jean Claude Schmit, Anthony D Harries

**Affiliations:** 1 Médecins sans Frontières, Operational Centre Brussels, Medical Department, MSF- Luxemburg, Luxemburg; 2 Institute of Tropical Medicine, Antwerp, Belgium; 3 Public Centre for Health Research and Central Hospital, Luxemburg; 4 International Union Against Tuberculosis and Lung Disease, Paris, France; 5 London School of Hygiene and Tropical Medicine, London, UK

## Abstract

Until now, we have all been desperately trying to run behind the HIV/AIDS epidemic and catch up with it, but despite all our efforts, the epidemic remains well ahead of us. In 2010, the antiretroviral treatment (ART) gap was about 60%, AIDS-related deaths were almost two million a year, and on top of these figures, for every one person started on ART, there were two new HIV infections. What is needed to change this situation is to think ahead of the epidemic in terms of the programmatic tasks we will be faced with and try to act boldly in trying to implement those tasks. From a programmatic perspective, we: a) highlight what needs to fundamentally change in our thinking and overall approach to the epidemic; and b) outline a number of key task areas for implementation and related operational research.

## Background

Until now, we have all been desperately trying to run behind the HIV/AIDS epidemic and catch up with it, but despite all our efforts, the epidemic remains well ahead of us. The annual estimates of people living with HIV/AIDS, new HIV infections and AIDS-related deaths since the dawn of the epidemic three decades ago provide undeniable justification to this statement. Although we are beginning to see some encouraging trends [1] we remain far from any epidemiological ideal of control.

In 2009, there were 5.25 million people receiving anti-retroviral treatment (ART) in low- and middle-income countries, which is a 40% coverage of those estimated to be in need of treatment [2]. However, with 2.6 million new HIV infections in 2009, for every person started on ART, there were two new infections, a sobering statistic that belies any claims that we are on top of this epidemic. AIDS continues to take its toll, with an estimated 1.8 million AIDS-related deaths in 2009 [1].

In summary, the backlog is high and the pipeline of new HIV/AIDS cases remains wide open. Given the enormity of the problem, it is not surprising that all the set implementation targets, such as the “3 by 5” Initiative [3] and “Universal ART access by 2010” [2] were not achieved. In the current state of affairs, the likelihood of achieving, by 2015, the United Nations Millennium Development Goal (MDG) [4] targets and the “Getting to Zero” target [5] (the three zeros: zero new HIV infections, zero discrimination and zero AIDS-related deaths) of the Joint United Nations Programme on HIV/AIDS (UNAIDS) seem highly unlikely.

What is needed is to look forward, think of appropriate strategies and follow these with bold action. Such action needs to be guided by embedding operational research into programmes. From a programme perspective, operational research has been defined as the search for knowledge on strategies, interventions or tools that can enhance the performance of health programmes in which the research is being conducted [6]. The authors of this paper have been involved with HIV/AIDS and related operational research at the programme level in sub-Saharan Africa for many years.

From a programme perspective, we: a) highlight what needs to fundamentally change in our thinking and overall approach to the epidemic; and b) outline a number of key task areas for implementation and related operational research.

## What needs to fundamentally change in our approach and thinking?

First, we need to imagine what the epidemic will look like in five to 10 years from now, as well as and the related programmatic challenges [7]. In this light, UNAIDS’s new innovative Treatment 2.0 [8] is one example of the kind of approach that is needed. This approach describes how ART and HIV/AIDS care delivery systems should evolve to become simpler and more accessible on a wider level so as to have a significant impact in closing the current treatment gap, as well as in preventing HIV infection. This approach could save up to 10 million lives and prevent millions of new infections [8].

Second, in light of the massive current gaps in both treatment and prevention, we must look beyond maintaining the status quo, which is not making an adequate “dent” in the epidemic.

Third, we need to overcome the current gaps of the traditional approach in documenting efficacy of new tools and strategies, such as randomized controlled trials, and the science of how to translate these into large-scale field implementation. Social science, in particular, is critical here and assists in contextual understanding of various issues. For example, randomized controlled trials have shown that circumcised adult men may have some protection against HIV infection [9,10], and it is now recommended that this surgical intervention be scaled up [11].

What is missing is a careful assessment of the potential consequences of large-scale, male circumcision initiatives that are currently taking place in six African countries [12]. It is quite possible that there are group or societal effects, including behavioural changes that may reduce, or enhance, the effect of male circumcision as a preventive strategy. The randomized controlled trial design avoids such contextual considerations, but in real life, these trials play an important role, especially when considering implementation on a large scale [13,14]. In particular, social science research on context-specific and gendered understanding and community messaging is likely to be vital in large-scale roll out.

Another example is the history of prevention of mother to child transmission of HIV (PMTCT), for which evidence and international recommendations exist [15]. However, contextual and feasibility challenges in sub-Saharan Africa have greatly affected uptake, with only about half of all HIV-infected pregnant mothers receiving antiretroviral drugs for PMTCT [1,12]. It is clear that gendered power relations within and beyond the household are likely to have a key role in whether or not mothers decide to engage with PMTCT programmes [16].

Finally, there is a need for new tools and new innovative strategies – even daring and bold action, things that most people might simply dismiss as being unfeasible – in different contexts.

## Thinking ahead on task areas for implementation and related operational research

Although not exhaustive, Table 1 highlights a number of key task areas for implementation. The content of this panel was developed through a two-day workshop conducted by Médecins sans Frontières (MSF), which included a wide group of stakeholders from sub-Saharan Africa, all of whom had been directly involved with implementation and scale up of HIV/AIDS programmes in the region for many years: programme managers working in ministries of health; district level implementers; academics; patient activist groups; international HIV/AIDS experts; and researchers. The identified task areas needing attention in the next five to 10 years included: sustaining long-term HIV/AIDS funding; offering care to the ever-growing cohorts needing ART; getting those still waiting for ART on treatment; monitoring patients in care; providing HIV care that is associated with a minimal risk of contracting tuberculosis (TB); investing in HIV prevention as a key to breaking the current epidemic and; building capacity for operational research. These and related operational research areas are discussed in the following pages.

**Table 1:** Specific task areas related to the HIV/AIDS epidemic and related operational research issues

**Task 1: Sustain long-term HIV/AIDS financing** Demonstrate how HIV/AIDS interventions contribute positively to improving healthcare services (directly or indirectly). Move from vertical to more integrated primary care delivery models. Show the positive impact of HIV/AIDS interventions at population level and clearly articulate how these link up with the achievement of the 2015 Millennium Development Goals and beyond. Perform economic analysis to show the economic gains of HIV/AIDS interventions on health services and beyond (HIV/AIDS interventions are cost effective?).
**Task 2: Think of how to offer care to the ever growing cohort on ART ahead of time** Enhance simplicity, efficiency and cost effectiveness of the delivery mechanism and adapt these to different contexts, for example: Design simpler and cheaper ART protocols that are less toxic (safer) and easier for patients and health services (for both first-line and second-line treatment). Pilot innovative models of delivery outside the health facilities (task shifting) and evaluate their effectiveness. Community-based models Expert patient-based models Workplace models. Find tangible solutions to the human resources shortage for health in Africa. Think of sustainable drug supply and commodity management chains that do not end up with drug stock outs Conduct social science research to better understand contextual issues influencing uptake of specific interventions.
**Task 3: Think of those still waiting to get onto ART** Offer ART earlier in line with revised WHO recommendations (41). Radically simplify ART eligibility assessment for patients in WHO stage 1 and 2 with point-of-care CD4 testing. Reduce high pre-ART attrition and test how to deliver specific packages of care (Cotrimoxazole preventive prophylaxis, , Isoniazid preventive therapy , Impregnated mosquito nets, nutritional support) that will support patients in pre-ART care and possibly keep them well and not yet needing ART.
**Task 4: Monitor cohorts of people with HIV in care** Develop and assess how to set up simple and robust monitoring and reporting systems to follow retention and attrition (pre-ART and on ART) of thousands and eventually millions of patients. Work out how to set quality indicators and acceptable quality thresholds for mass scale up. Use point-of-care viral load tests for monitoring adherence, as well as deciding the optimal time to switch to second-line therapy. Use available and new technology to boost adherence, e.g., telephones and online tools.
**Task 5: Provision of HIV care that is associated with a minimal risk of tuberculosis** Determine how best to implement TB case finding, infection control (air ventilation and patient flow organization) and isoniazid preventive therapy. Determine how to monitor these interventions.
**Task 6: Invest in HIV prevention as this is a key to breaking the current epidemic** Find innovative ways of improving HIV testing and knowledge of HIV status. Assess feasibility of male circumcision on a large scale: Through simpler and safer techniques Integrating circumcision into preventive services (e.g., WHO’s Expanded Programme on Immunization) Enhancing community and civil society acceptance through partnerships. Pilot the feasibility and effectiveness of ART for prevention in the general population and high-risk groups. Radically simplify the PMTCT approach and protocol for health service providers and the mother (e.g., a one-pill-a-day standardized approach). Assess feasibility of pre-exposure prophylaxis in high-risk groups and discordant couples. Understand the societal factors influencing uptake through social science research in relevant areas.
**Task 7: Build capacity for conducting operational research: testing out new models of training** Try out and assess different types of training models and curricula that are performance and/or output based.

### Sustaining long-term HIV/AIDS funding

Over recent years, there has been growing evidence of donor fatigue and what seems like a retreat from HIV/ AIDS funding by major donors. In 2010, the Global Fund to Fight AIDS, Tuberculosis and Malaria received US$11.7 billion in pledges compared with the $20 billion it had said it needed to meet the target of Universal Access. The United States-funded US President’s Emergency Plan for AIDS Relief programme, which supports at least half of all people on HIV/AIDS treatment in developing countries, has flat-lined funding for the third year in a row [17].

These are worrying signs, and economic recession is not the only culprit. Although not formally stated, issues of concern to donors include the fact that HIV/AIDS interventions have predominantly been vertical, with limited evidence of wider benefits to health systems [18,19]. There is some evidence demonstrating positive benefits of HIV/AIDS interventions [20]. A few specific examples include: the impact of ART in keeping health workers alive, particularly in sub-Saharan Africa [21]; the positive population impact of HIV/AIDS interventions in reducing crude mortality [22]; and community-wide benefits in reducing the incidence of such diseases as TB [23-25] and malaria [26,27]. More examples of this type of operational research are needed to provide evidence of a broader impact both within and outside of the health sector, and this should help convince donors to invest in HIV/AIDS as a integral and worthwhile strategy towards achieving the health-related MDG targets [4]. In particular, operational research demonstrating a positive impact on maternal and child health services would be of great value [28].

### How to offer care to the ever-growing cohort on ART?

More than five million people are on ART in low- and middle-income countries, and this case load is expected to double or triple in the next five to 10 years. The daunting challenges include: retaining and managing such patients [29]; providing an uninterrupted supply of drugs; ensuring adherence to medication; monitoring drug-related side effects; and detecting and managing drug resistance. The underlying problem is that the increase in case loads and the consequent challenges occur in situations where there are serious shortages of health workers and weak health infrastructure. Sub-Saharan Africa, for example, has a shortfall of almost five million health workers, more than double the numbers working today [30,31].

An additional problem is that most delivery models of care are labour intensive, with doctors and nurses playing a central role in service delivery and healthcare management [32,33]. In addition, most ART and support services are run from hospitals, but most of these hospitals are located in urban centres that patients from distant rural and semi-urban settings cannot access because of high transport and opportunity costs [34].

Encouraging evidence from decentralized, home-based models involving empowerment of non-clinical workers is emerging [35-37]. Importantly, a cluster randomized trial in Uganda demonstrated the cost effectiveness of home-based HIV care using non-clinical workers [36]. Similarly, recent encouraging evidence from rural Mozambique showed that self-forming groups of patients successfully conducted four vital activities: they facilitated monthly ART distributions to their group members in the community; they provided adherence and social support; they monitored outcomes; and they ensured that each group member underwent a clinical consultation at least once every six months [37]

Several point-of-care CD4 and viral load tests are either on the market or in development, and offer an opportunity to decentralize monitoring to the patient level [38]. For example, people with diabetes can check their blood glucose levels almost anywhere in the world with point-of-care tools. If a mother is worried that her child has a fever, she can use a thermometer to check if this is the case. Similarly, we need a simple test for monitoring viral load and CD4 counts that can be used at home, something akin to a “dipstick” that can be easily read and interpreted by lay people.

We already do have a single-tablet fixed-dose combination for first-line ART (Atripla), and this can help radically simplify the treatment protocol. If this can evolve further into a pill that has no side effects and that has a high barrier to the development of resistance, it would be ideal. Further simplification could also be achieved if new regimens could be developed that allow greater spacing, for example, once-a-week treatment. This could ease stock and supply management as well as may be more cost advantageous. A decreased frequency of interaction with healthcare providers will free up HIV/ AIDS-dedicated human resources, lower out-of-pocket costs, such as transport fees for the care seeker [8], and would bring obvious advantages to the task of simplifying drug and commodity management.

In terms of operational research, what is needed is to implement and assess the feasibility and effectiveness of delivery models for scale up of ART that move away from hospitals to communities and from clinical to non-clinical staff [32,39,40]. There is also a need to decentralize point-of-care tools, such as CD4 and viral load tests, to the patient level and assess if self-testing can be used responsibly. Finally, there is a need to find ways of radically simplifying treatment protocols and assessing the impact that this has on patient and programme outcomes.

### Getting those still waiting for ART onto treatment

At present, about six out of every 10 persons who need ART fail to access treatment [12]. When patients eventually get to the ART clinic, they are often weak, with advanced immune suppression, and despite treatment for opportunistic infections, a high proportion die early [8]. Reducing the waiting list for ART will need fast tracking of patients with simpler and less labour-intensive models and starting patients on ART earlier (with CD4 counts equal to or less than 350 cells/mm^3^ or less), as has been recommended by the World Health Organization (WHO) [41].

For example, a randomized controlled trial in Haiti showed convincingly that early initiation of ART was associated with a lower risk of death and incident risk of TB [42]. Operational evidence from Lesotho adds to this knowledge, showing that providing ART earlier leads to a 68% reduction in deaths, a 27% reduction in new opportunistic diseases, a 63% reduction in hospitalization, and a 39% reduction in people defaulting from care [43].

The use of WHO staging, as now recommended by WHO, avoids the need for CD4 tests to determine ART eligibility in those with advanced clinical disease (WHO stages 3 and 4) and thus shortens the road to ART. The challenge remains for patients in earlier clinical stages (WHO stages 1 and 2) who still need CD4 counts to assess their ART eligibility [41]. Finally, early infant diagnosis remains problematic, and in 54 reporting countries, only 15% of children born to HIV-infected mothers were tested for HIV in the first two months of life [12].

In terms of operational research, interesting challenges would include assessing the feasibility and effectiveness of a point-of-care CD4 test at community or patient levels as a gateway to facilitate access to ART for patients in WHO stages 1 and 2 [38]. Finding ways of delivering specific packages of care (e.g., cotrimoxazole prophylaxis, isoniazid preventive therapy, nutritional support) to support patients in pre-ART care and to prevent pre-ART attrition is required [44]. Finally, there is a need to develop and assess the feasibility of simple-to-use and accurate HIV diagnostic tools for infants with the aim of improving early HIV diagnosis in this highly vulnerable group.

### Monitoring cohorts in care

Current monitoring systems are geared towards following patients for retention and adherence over time. The sources of information for such monitoring are patient cards and registers. The work involves manual, labour-intensive reviews, which are unsustainable in the medium to longer term as reporting cohort outcomes involves hours of manual work, sifting through individual patient cards and registers of hundreds if not thousands of patients. Radical simplification is needed [45,46].

Encouraging ways forward include, for example, the use of robust electronic touch screen systems with automated cohort reporting [47,48]. The use of existing common technology, such as mobile phone messaging in Kenya to enhance adherence, reduced the need for patients to physically return to health facilities and proved effective in enhancing ART adherence and viral load suppression [49]. In terms of operational research, there is a need to explore and further field test the potential of mobile phone technologies, including smart phones, and other innovative approaches linking the ever-expanding technology with healthcare interventions. Piloting new and effective ways of moving from district-level reporting to facility-based monitoring and reporting, so that individual health facilities are able to manage and use their data in an effective manner, is also much desired.

### Provision of HIV care that is associated with a minimal risk of tuberculosis

WHO recommends the implementation of a package of the Three “I”s (isoniazid preventive therapy, intensified case finding and infection control) as being essential to effective HIV and TB care that is safe from nosocomial TB transmission [50,51]. Despite good evidence of the benefits of this package, we have been far too slow in the application of knowledge into practice. The implementation gaps remain wide, with only 2.5% of 1.9 million eligible individuals screened for TB and only 8.7% of 16 million people placed on isoniazid preventive therapy. There are no global data on infection control [52]. Ways forward to enhance feasibility and uptake of each of these components are urgently needed [52,53].

### Investing in HIV prevention strategies as a key to breaking the current epidemic

Prevention is key to breaking the current epidemic as this will close the pipeline of new cases that adds to existing case loads and mortality. Simulations have consistently shown that in the longer term, the most important driver of HIV prevalence and HIV case load is present-day HIV incidence. Halving of HIV incidence leads, with a eight-to 10-year lag, to halving the number of people needing to start ART, and considerably lower overall HIV prevalence [54] The first challenge is that only about 40% of people living with HIV currently know their HIV status. Second, there is a need to find feasible and acceptable ways of rolling out biomedical interventions, such as male circumcision at the community level [11,13,14]

Third, there is the dramatic impact of ART on prevention through viral suppression [8,55] A recent study showed that ART used in discordant couples reduces HIV transmission (between couples) by 92% [56]. Mathematical modelling has also suggested the potential impact of offering annual universal voluntary HIV testing and counselling, followed by immediate ART, irrespective of clinical stage or CD4 counts. Such a strategy would reduce the transmission rate (Ro) to <1 (Ro being defined as the number of secondary infections resulting from one primary infection in an otherwise susceptible population). As a consequence, the incidence of HIV would be reduced to less than one case per thousand persons per year within 10 years of full implementation of the strategy and the prevalence of HIV to less than 1% within 50 years [57-59].

Fourth, PMTCT remains a major challenge and finding ways forward in increasing access and uptake is urgently needed. Even the current WHO protocol [15] is complicated; what is needed is a radical simplification of the PMTCT approach and protocol for both the provider and the mother. Finally, a randomized, double-blind multi-country trial among HIV-negative men and trans-gender women who have sex with men showed that pre-exposure prophylaxis with two oral ART drugs (tenofovir and emtrictabine) was associated with a 44% reduction in HIV incidence compared with placebo, the prophylactic effect being strongly correlated with detectable blood levels of the two drugs [60,61]. The CAPRISA 004 trail also demonstrated the effectiveness and safety of a 1% vaginal gel formulation of tenofovir for the prevention of HIV acquisition in women [62].

In terms of operational research, there is an urgent need to find ways of increasing knowledge of HIV status by increasing the uptake of HIV testing and linkages to care [8] The feasibility and acceptability of integration of circumcision into routine preventive activities, such as WHO’s Expanded Programme on Immunization, or as stand-alone services needs to be explored. In terms of ART for prevention at the community level, there is an urgent need to think around the challenges of acceptability and feasibility of this approach in the general population and among high-risk groups [57-59]. For PMTCT, a one-pill-a-day ART, a standardized approach that covers both prevention of transmission and early treatment of the infected mother, should be tried. Finally, feasibility of implementing pre-exposure prophylaxis should be explored under operational conditions, and to save on costs, the efficacy of pre-exposure prophylaxis only at the time of sexual exposure should be examined. Combining implementation and social science research in all these areas is vital.

### Building capacity for conducting operational research: testing out new models of training

Programmes in low-income countries and, particularly in sub-Saharan Africa, need to develop the capacity to conduct operational research [6,30,63]. Some of the programmatic challenges include inadequate or irrelevant research questions, lack of time and opportunity, lack of research infrastructure, and lack of knowledge about how to translate research findings into policy and practice [6]. In particular, capacity to conduct and publish research is seriously lacking [64]. Programme-embedded models with strong on-the-job mentorship are urgently needed. Training models will need to take programmatic hurdles into consideration, to be performance and output linked, and able to deliver in a relatively short time. Two examples of such approaches include the International Union Against Tuberculosis and Lung Disease-MSF approach to sustainable operational research and that offered by the US Centers for Disease Control and Prevention [65-67]. Operational research is also needed to evaluate the existing models and tailor them to ensure outputs.

## Summary

Turning the tide of the devastating HIV/AIDS epidemic will require us to think ahead about the challenges with regards to policy, care and prevention that we will be facing in the years to come. It is beholden upon us both as implementers and researchers to act now through bold action and operational research so that will be able to face up to current and impending tasks related to the epidemic.

## Competing interests

The authors have no competing interests to declare.

## Authors’ contributions

RZ and ADH wrote the first draft manuscript which was then critically reviewed and improved by WvD, VA and JCS. All co-authors were involved with the final version and have approved it.
